# Effects of W/O Nanoemulsion on Improving the Color Tone of Beijing Roast Duck

**DOI:** 10.3390/foods12030613

**Published:** 2023-02-01

**Authors:** Wendi Teng, Xinshuo Yao, Jingyi Li, Jinpeng Wang, Jinxuan Cao

**Affiliations:** 1 School of Food and Health, Beijing Engineering and Technology Research Center of Food Additives, Beijing Technology and Business University, Beijing 100048, China; 2 College of Food and Biological Engineering, Chengdu University, Chengdu 610106, China

**Keywords:** W/O emulsion, stability, Maillard reaction, Beijing roast duck, color tone

## Abstract

Traditional Beijing roast duck is often brushed with a high concentration of maltose solution (15% *w*/*v*) and shows ununiform color after roasting. A novel W/O nanoemulsion was applied to improve the color tone of Beijing roast ducks and, meanwhile, reduced the amount of sugar. For the W/O emulsion, 3% (*w*/*v*) xylose solution as the aqueous phase, soybean oil as the oil phase, and polyglycerol polyricinoleate (PGPR) and whey protein isolate (WPI) as co-emulsifiers were fabricated by high-pressure homogenization. Particle size measurement by Zetasizer and stability analysis by Turbiscan stability analyzer showed that WPI as co-emulsifier and internal aqueous phase at pH 9 decreased the droplet size and improved the emulsion stability. In addition, by color difference evaluation, the W/O nanoemulsion improved the Maillard reaction degree and color tone of Beijing roast duck. The molecular structure and key composition of pigments on the surface of Beijing roast duck skins were also identified and characterized by UV–vis spectroscopy and UHPLC-MS. This study creatively offers theoretical guidance for increasing applications of W/O-nanoemulsion-based Maillard reaction in the roast food industry, especially for the development of reduced-sugar Beijing roast duck with uniform and desired color satisfying consumers’ acceptance and marketability.

## 1. Introduction

Beijing roast duck is one of Beijing’s unique and classical cuisines. A hundred years of development makes it popular around the world for its attractive color, palatable flavor, and special texture, as it is roasted in a special wood oven at a high temperature. During roasting, the Maillard reaction, a type of non-enzymatic reaction, occurs when the carbonyl group in the reducing sugars reacts to the amino group of amino acids, polypeptides, or proteins and produces pigmentation and aromatic compounds, stimulating appetite and affecting multiple food quality parameters [1,2,3,4]. Pigmentation through the Maillard reaction is attributed to melanoidins. Various pigments accumulatively turn into the color, even though the amounts of individual pigment are low. The reaction conditions, such as types and concentrations of reactants, temperature/time combination, pH, and matrix, influence the formation of the pigments, showing that the color tone of the reaction solution or food products is changeable [5,6]. Traditional roast duck preparation often includes six processes: slaughtering, air pumped into the duck, boiling water poured on it, sugaring, drying, and roasting. What determines its color and crispness is the process of sugaring. Beijing ducks are brushed twice with about 15% maltose solution (1 kg maltose dissolved in 5–7 L of water) on the skin. However, after roasting, the color of Beijing roast ducks in many restaurants is not quite uniform; it is dark in some parts of the duck skin but light in other parts, which severely influences people’s desires and appetite.

Water-in-oil (W/O) is an emulsion form of many lipid-rich systems which could be applied as delivery carriers for bioactive loads, antioxidant protection, controlled slow release, flavor maintenance, and so on [7,8]. However, W/O emulsions are thermodynamically unstable colloidal dispersions comprising oil, emulsifiers, and water [9]. Various emulsifying factors, such as temperature, pH, and ionic strength, can affect emulsion stability, which is a critical step for industrial utilization [10]. Nanoemulsions with particle sizes between 1 and 100 nm could improve the physicochemical properties, preventing gravitational separation and particle aggregation [11]. Moreover, a smaller droplet size could enlarge the droplet surface area, thus enhancing the related functionality [12].

In this paper, we hypothesized that a W/O nanoemulsion with a droplet size less than 100 nm could be homogeneously brushed and distributed on the duck skins. As a result, it would have a better effect on improving and homogenizing the color tone of Beijing roast ducks than the maltose solution. For the W/O emulsion, a xylose solution was chosen as the aqueous phase, soybean oil as the oil phase, and polyglycerol polyricinoleate (PGPR) and whey protein isolate (WPI) as co-emulsifiers. There are several theoretical bases for the hypothesis. Firstly, the fat content of Beijing duck skin is about 33.4–42.8% [13]. Such a high amount of fat makes the maltose solution hard to evenly distribute on the duck skin. In contrast, with the high-viscosity soybean oil as the continuous phase, the W/O emulsion could be uniformly brushed and distributed on the skin. Secondly, xylose is a reducing monosaccharide that cannot be digested and absorbed by the gastrointestinal tract. Many studies have reported its effects on regulating blood glucose, insulin secretion, and anti-obesity [14]. Therefore, xylose is a better choice than maltose for people who want to have Beijing roast duck but worry about ingesting too much sugar at the same time. Thirdly, PGPR, generally recognized as safe (GRAS), is an effective lipophilic emulsifier [15] which could be widely used to stabilize W/O emulsions [16]. However, one emulsifier can easily fall from the droplet surface through physical adsorption, resulting in poor stability [17]. WPI, mainly composed of *α*-lactalbumin, *β*-lactoglobulin, bovine serum albumin, and immunoglobulins, contains all the essential amino acids and is a natural active surfactant that meets consumers’ expanding demand for healthy and environmentally friendly food [18,19,20]. In this study, a combination of PGPR and WPI was used as emulsifiers to better stabilize the W/O emulsion. Last, xylose and WPI in this W/O emulsion system are also the substrates of the Maillard reaction, which could help improve the degree of the Maillard reaction during roasting.

The main goal of the research was to determine whether the W/O emulsion could improve the color tone of Beijing roast ducks. Therefore, a W/O-nanoemulsion-based Maillard reaction was established in the work. The impact of pH and emulsifiers on the droplet size and stability of the W/O nanoemulsion was determined. Additionally, the effects of the W/O nanoemulsion on Maillard reaction degree, color tone, and browning intensity of Beijing roast ducks were evaluated. The molecular structure and key composition of pigments on the surface of Beijing roast duck skins were also identified and characterized. This study focused on a W/O-nanoemulsion-based Maillard reaction suitable for utilization in food and offers new ideas in developing effective strategies to reduce the amount of sugar without adversely affecting the desirable physicochemical attributes or sensory profile.

## 2. Materials and Methods

### 2.1. Materials

Non-genetically modified organism Grade I soybean oil purchased from COFCO Co. (Beijing, China), D-xylose (purity > 99%) from Mreda Tech Co. (Beijing, China), polyglycerol polyricinoleate (PGPR, HLB = 3) from Daheng Food Tech Co. (Zhengzhou, China), and whey protein isolate powder (WPI, purity > 80%) from Yuanye Biotech Co. (Shanghai, China) were used in producing the nanoemulsion. D-(+)- maltose (purity > 99%) was purchased from Macklin Biochemical Technology Co. (Shanghai, China). Ultrapure water obtained from the Milli-Q Plus apparatus (Millipore, Billerica, MA, USA) was used in the formulations of nanoemulsions. Beijing ducks were purchased in a local grocery store. All other chemicals used in this study were of analytical grade from Sinopharm Chemical Reagent Co. (Shanghai, China).

### 2.2. Preparation of W/O Emulsion

The emulsion was prepared by a previously reported method [21]. Before emulsification, 3% (*w*/*v*) of PGPR was mixed with soybean oil at 50 °C to form the oil phase. A total of 1% (*w*/*v*) of WPI and 3% (*w*/*v*) of xylose was dissolved in ultrapure water to form the aqueous phase, and the pH value was adjusted to 5, 7, or 9 respectively. The ratio of the water phase and the oil phase was 3:7. The coarse emulsion was prepared by adding water dropwise to the oil phase using IKA T25 digital Ultra-Turrax (Schwabach, Germany) by high-speed shear homogenization at 15,000 rpm for 10 min. The mixture was then homogenized and passed through a high-pressure valve homogenizer (15MR-8TA, APV Gaulin Inc., Wilmington, MA, USA) at 20 MPa. The resulting W/O emulsions were kept at 25 °C in a temperature control incubator.

### 2.3. Measurement of Emulsion Droplet Size

Dynamic light scattering (DLS) with a Zetasizer Nano ZS90 (Malvern Instruments, UK) was applied to measure the droplet size and polydispersity index (PDI) of the W/O emulsion ((η = 40 mPa s at 25 °C, RI = 1.46) [22]. Soybean oil was first treated with ultrasound for 5 min to prevent the formation of bubbles. Samples were then diluted to the ratio of 1:40 with soybean oil to ensure the free Brownian motion of droplets. Samples were put in 1 × 1 polystyrene cuvettes. Measurements were performed just after emulsion preparation at 25 °C.

### 2.4. Stability Analysis

Emulsion stability was determined with Turbiscan Lab (Formulaction, France). A total of 20 mL of freshly prepared emulsions were placed in flat-bottomed glass test tubes (43 mm height) and scanned at 0, 0.5, 1, 2, 4, 8, and 17 h, respectively. The tubes were then stored at 25 °C for 7 days and scanned once at the same time every day until day 7. Emulsion destabilization was analyzed using backscattering (BS) profiles at different sample height. A sample at 0 mm of height corresponds to the bottom of the test tube. The Turbiscan stability index (*TSI*) was used to determine the stability of the entire dispersion system and calculated as follows:

(1) $$TSI=\underset{j}{\sum }|scan_{ref}\;\left(h_{n}\right)-scan_{i}\;\left(h_{i}\right)|$$

*Scan_ref_* and *scan_i_* stand for the backscattering value at the initial stage and after 7 days’ storage, respectively. *h_n_* stands for the given height in the measuring cell. *TSI* is the sum of all the scan differences through the tube.

### 2.5. Production of Beijing Roast Duck

A total of 2000 ± 300 g of 42-day-old Beijing ducks were used and processed according to traditional techniques at Jinbangyuan restaurant in Beijing. Air was pumped into the duck between its skin and subcutaneous tissue, making the duck look plump. Then, boiling water was poured over the duck three times, and it was dried for 12 h at 4 °C. 15% (*w*/*v*) maltose solution, 3% (*w*/*v*) xylose solution, or the W/O emulsion was brushed on the duck skin, respectively, followed by drying for 2 h at 4 °C. Finally, the ducks were hung in the traditional oven for roasting at 230 °C for 30 min. After roasting, duck skins were separated, cut into small pieces, wrapped with aluminum foil, and stored at −20 °C until analysis.

### 2.6. Color Measurement of Beijing Roast Ducks

Color differences of Beijing roast ducks were measured via a colorimeter (Konica Minolta, Tokyo, Japan). After the roast ducks cooled down, the CIE color parameters (lightness (L*, light/dark), redness (a*, red/green), and yellowness (b*, yellow/blue)) were immediately evaluated at the belly of each duck with forty random points.

### 2.7. Extraction and Purification of Pigments

The pigments were extracted based on the method in previous research [23]. Briefly, 2 g of minced duck skin was dissolved in 20 mL of 85% ethanol, which was then subjected to ultrasonication at 400 W at 25 °C for 30 min. The extracting solution was centrifuged at 6000× *g* for 15 min. The supernatant was filtered by a neutral filter paper, condensed by rotary evaporation at 60 °C, and finally, the pigments were obtained.

### 2.8. UV–Visible Spectra Analysis

The pigment samples were dissolved in ethanol. The UV–VIS absorption wavelength was determined via UV-2600 spectrophotometer (Shimadzu, Tokyo, Japan) at the spectral range of 200 to 800 nm.

### 2.9. UHPLC/MS Analysis

The pigment solution was filtered by a syringe filter (Anpel Laboratory Co., Shanghai, China) for the UHPLC test. The separation and identification of pigments were carried out via a Nexera UHPLC LC-30A system (Shimadzu, Tokyo, Japan) and a Triple TOF5600+ mass spectrometer (AB Sciex, Foster City, CA, USA) as well as a Sepax GP-C18 Column (1.8 µm 120 Å 2.1 mm × 150 mm). The mobile phase included 0.1% formic acid (A) and acetonitrile (B). The procedure of gradient elution using UHPLC was as follows: 0 min (A:B = 95:5), 1 min (A:B = 30:70), 17 min (A:B = 0:100), and 19 min (A:B = 95:5), until 21 min. The flow rate was 0.3 mL/min. The mass spectrometer was operated in both negative and positive ion modes. The acquisition range was from 50 to 1000 m/z.

### 2.10. Statistical Analysis

All the experiments in the present study were carried out at least in triplicate, and data were shown as means ± standard deviations (SD). Significant differences (*p* < 0.05) between means were evaluated by one-way ANOVA, followed by Duncan’s multiple-comparison test using SPSS software (version 20.0, IBM Inc., Chicago, IL, USA).

## 3. Results and Discussion

### 3.1. Average Diameter and PDI of W/O Emulsion

**Figure 1:**
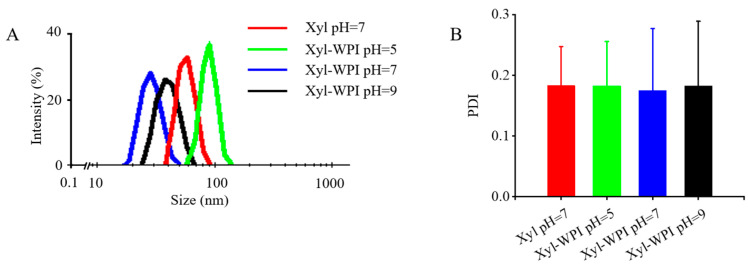
Particle size distribution and PDI value of different W/O emulsions. (**A**) Particle size distribution and (**B**) PDI value. Xyl W/O: Emulsion stabilized by PGPR; Xyl-WPI W/O: Emulsion stabilized by PGPR and WPI; pH refers to the pH value of internal aqueous phase.

Particle size is an essential value influencing the stability and some other essential characteristics of an emulsion [24]. Before the addition of WPI into the internal aqueous phase, the average droplet size of the W/O emulsion was 57.11 nm (Figure 1A). After adding WPI into the internal aqueous phase at pH 5, close to the isoelectric point of WPI, it showed larger mean droplet diameter (136.8 nm), which was similar with the results in previous research [25,26]. However, adding WPI into the internal aqueous phase at pH 7 or 9 resulted in a smaller size (28.74 nm and 39.53 nm) compared with that in the W/O emulsion without WPI addition. The result was consistent with a study showing that an increased amount of WPI in the internally dispersed phase decreased the particle size and enhanced the emulsion stability [27]. All the emulsions had only one narrow intensity peak and were on the nano-range scale. This would be attributed to the high-pressure homogenization, which produces intense disruptive forces, resulting in big droplets breaking into smaller ones [12]. Moreover, smaller droplet sizes on the nano-range scale bring an enhancement in stability [12].

PDI is another parameter indicating the heterogeneity of particle distribution. Values near 0 suggest homogenous distributions, while values near 1 suggest heterogeneous ones. As shown in Figure 1B, the PDI values of all the emulsions were ≤0.28, indicating the even distribution of droplets in freshly made emulsions after homogenization.

### 3.2. Emulsion Stability

**Figure 2:**
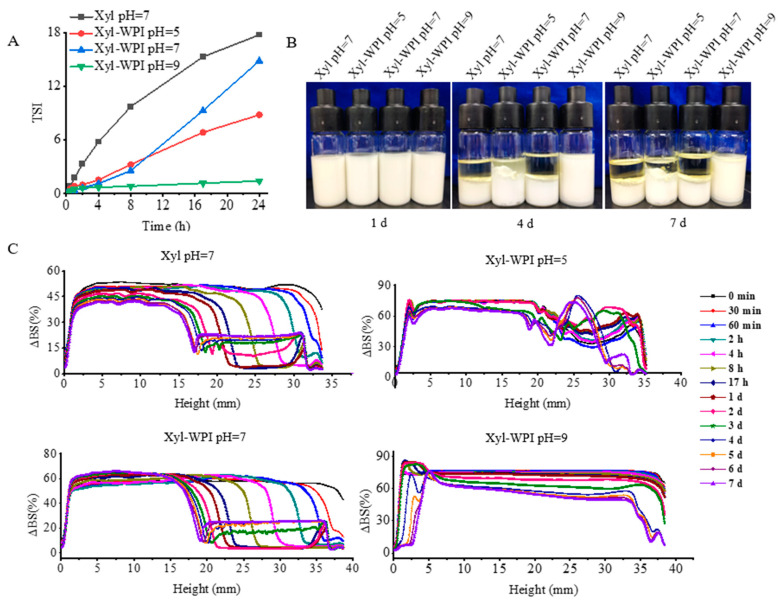
Global stability of different W/O emulsions during 7 days of storage at 25 °C. (**A**) *TSI* values of emulsions within 24 h. (**B**) The appearance of emulsions kept at day 0, 4, and 7. (**C**) Backscattering profiles (ΔBS) of emulsions within 7 days. Xyl W/O: Emulsion stabilized by PGPR; Xyl-WPI W/O: Emulsion stabilized by PGPR and WPI; pH refers to the pH value of internal aqueous phase.

Though W/O emulsions show great potential, they are not widely applied, owing to their thermodynamic instability [28,29,30,31]. A stable W/O emulsion depends on the interaction between the emulsifier and oil phase to form a rigid oil–water interface by the tight anchoring of the emulsifier to the oil phase. In addition, smaller water droplets reduce the coalescence due to the lower collision efficiency, thus improving the stability [28]. This study evaluated if WPI as a co-emulsifier and internal water phase at different pH values could influence the stability. The Turbiscan stability analyzer was used to measure the stability, which was often evaluated by the change of *TSI* and backscattered light intensity (ΔBS). When the *TSI* value increased, the stability decreased. The kinetic stability of different W/O emulsions within 24 h was compared and shown in Figure 2A. Data showed that the *TSI* value of the Xyl W/O emulsion increased, suggesting that the W/O emulsion prepared without WPI tended to be unstable. However, when WPI was added into the internal aqueous phase, the *TSI* value showed a slow increase, suggesting that WPI enhanced the stability. Especially for the internal aqueous phase at pH = 9, the *TSI* value remained nearly unchanged within 24 h. In order to better compare the stability over 7 days of storage, ΔBS profiles of the W/O emulsions were evaluated in Figure 2C. The ΔBS profiles at the top of the emulsion samples continuously decreased, indicating an oil-off phenomenon, therefore resulting in the formation of a more transparent upper phase. The phenomenon could also be seen in Figure 2B, showing that within the first 4 days, all the emulsions exhibited a transparent layer at the top of the tube, except for the Xyl-WPI W/O emulsion at pH 9. Instead, the ΔBS profiles at the bottom of that emulsion slightly increased, suggesting that a slight phase separation occurs in the Xyl-WPI W/O emulsion at pH 9. Based on the data of global stability, the WPI and internal water phase at pH = 9 largely enhanced the stability. This was perhaps because WPI formed an extra coat around the internal aqueous droplets, and the coat on the surface would hamper water droplets penetration during the fabrication of the W/O emulsions, thus preventing the instability. Additionally, WPI has few net charges and fewer protein molecules accumulated at the oil–water interface under pH conditions close to the isoelectric point, which weakened the electrostatic and steric repulsions of the interface film, resulting in relatively low emulsion stability [32].

### 3.3. Color Difference Analysis

**Figure 3:**
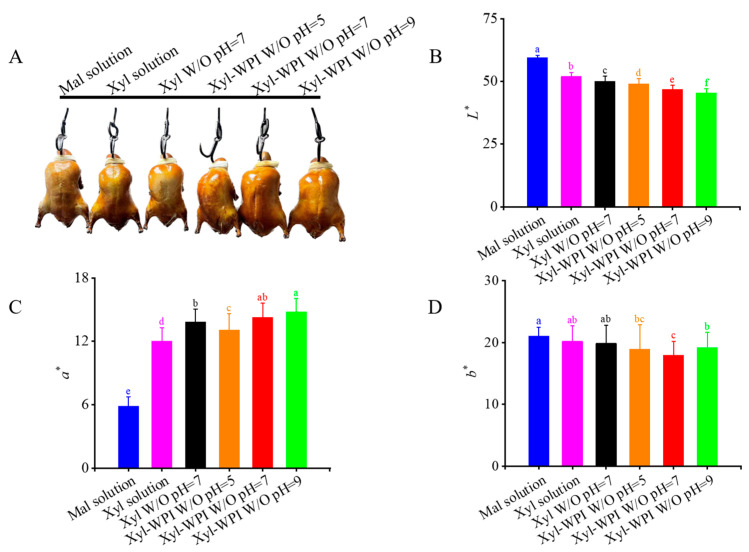
Color differences of Beijing roast duck upon different treatments after roasting for 30 min at 230 °C. (**A**) The picture, (**B**) L* values, (**C**) a* values, and (**D**) b* values. Solution or emulsion was brushed on the duck skin before roasting. Different letters (a–e) indicate statistically significant differences (*p* ≤ 0.05), according to ANOVA (one-way) and the Tukey test. Mal solution: 15% maltose solution; Xyl solution: 3% xylose solution; Xyl W/O: Emulsion stabilized by PGPR; Xyl-WPI W/O: Emulsion stabilized by PGPR and WPI; pH refers to the pH value of internal aqueous phase.

Color is often the first sensory characteristic and influential attribute which directly affects consumer preferences and desires. The formation of brown pigments contributes to the development of color. The color differences of Beijing roast ducks are presented in Figure 3. As shown in Figure 3A, after roasting, the duck in the maltose solution group showed the lightest color. In comparison, the xylose solution group and the Xyl W/O emulsion group showed a darker color, but it was not evenly distributed across the skin. However, the ducks in the Xyl-WPI W/O emulsion groups showed surprisingly attractive and uniform color, indicating that it optimized the Maillard reaction and improved the color tone.

In order to further analyze the color differences, the degree of color change was determined by L* (lightness), a* (redness), and b* (yellowness). The L* value is often used as an index of roasting degree, indirectly reflecting the degree of the Maillard reaction [33]. As shown in Figure 3B, the L* value in the Xyl-WPI emulsion groups significantly decreased, suggesting that the Xyl-WPI W/O emulsions greatly decreased the brightness. In Figure 3C, the increase in a* values indicated the formation of more brown pigment via the Maillard reaction [34]. For the b* value, there was no significant difference between the xylose solution group and the emulsion groups (Figure 3D). Among all the groups, the Xyl-WPI emulsion at pH = 9 showed the lowest L* but highest a* value, indicating the improved reaction rate and formation of the Maillard reaction products. It was perhaps because the higher pH offered suitable situations for the molecular rearrangement of sugar and facilitated the nucleophilic addition reaction [35]. Moreover, Amadori compounds would form 1, 2-enolization at pH 8 and tend to react with 2, 3-enolization at pH 9.7 [36]. Furthermore, WPI not only acted as the emulsifier to stabilize the emulsion, but also functioned as the substrate to enhance the Maillard reaction. Therefore, the degree of Maillard reaction and color tone of Beijing roast duck was deepened with WPI and increasing pH. Lotfy et al. also evaluated the effect of pH on color by a quinoa protein hydrolysate-xylose Maillard model, concluding that increasing the pH value gave rise to increasing browning intensity of the Maillard reaction products [37].

### 3.4. UV–Vis Spectroscopy Analysis of Pigment

**Figure 4:**
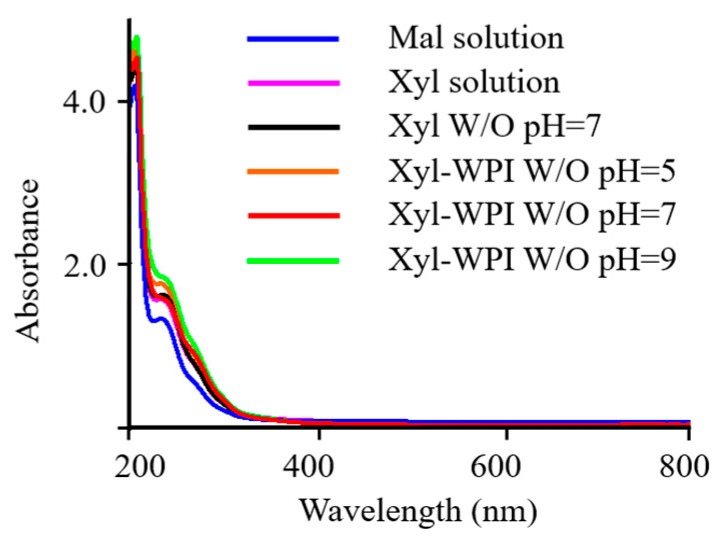
UV–vis spectroscopy of pigments from Beijing roast duck upon different treatments. Mal solution: 15% maltose solution; Xyl solution: 3% xylose solution; Xyl W/O: Emulsion stabilized by PGPR; Xyl-WPI W/O: Emulsion stabilized by PGPR and WPI; pH refers to the pH value of internal aqueous phase.

UV–vis spectroscopy of pigment containing structural information is shown in Figure 4. It shows that the absorption occurred between 200 and 400 nm, with a maximum absorption at 205 nm and a shoulder at about 235 nm, and the absorption decreased to the visible region. It was consistent with the data published by Hong et al., which showed a gradually increased absorption from 200 to 400 nm for brown pigments in Chinese sugar-smoked chicken [23]. The UV–VIS absorption spectrum of the pigment at 205 nm was also similar to that of melanin pigmentation [38]. Moreover, it could be seen that the absorbance in W/O nanoemulsion groups was higher than that in the solution groups, and Xyl-WPI emulsion at pH = 9 showed the highest absorbance at 205 nm, perhaps owing to accumulated pigmentation via the Maillard reaction.

### 3.5. Identification of the Key Constituent of Pigments by LC-MS Analysis

UHPLC is widely used for the successful separation of components from the Maillard reaction. TOF-MS is suitable for the measurement of exact molecular masses [39]. To characterize the composition of brown pigment, TOF-MS with ESI was used to identify the brown pigment of the duck skin in the Mal solution group, the Xyl solution group, and the Xyl-WPI W/O pH 9 group. Brown pigment was found to be a mixture containing various chemical compounds. MS information was extracted and analyzed via the software MS-DIAL 4.70, and the peak information was searched and compared within three databases, MassBank, Respect, and GNPS (14,951 records in total). About 80 compounds were found by MS. Among them, 17 compounds were found in the Xyl-WPI W/O pH 9 goup but not in the other two groups. After the analysis, three compounds were focused on.

**Figure 5:**
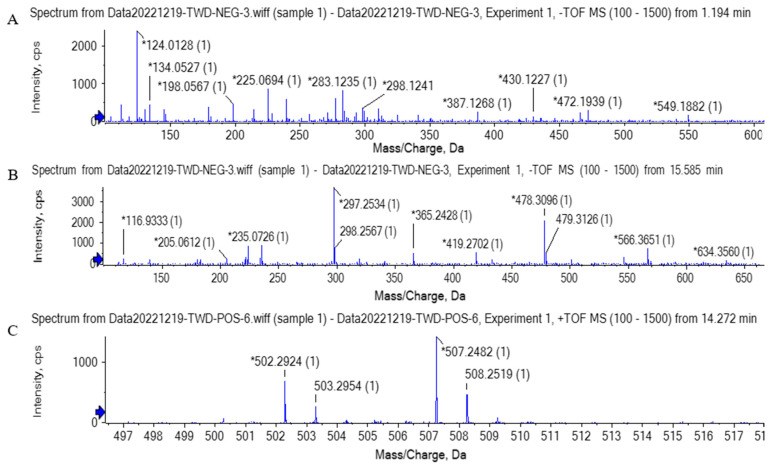
The primary mass spectrogram (MS) of compounds. (**A**) Compound 1 (*m/z* = 283.12), (**B**) compound 2 (*m/z* = 365.24), (**C**) compound 3 (*m/z* = 502.29).

As shown in Figure 5A, the quasi-molecular ion peak was obtained at m/z 283.12, which was separated at 1.194 min. The possible chemical formula was C_14_H_20_O_6_, which was speculated to be phenylethyl 2-glucoside. Glycosylation happens in the Maillard reaction. Reducing sugar and the amino group of protein first forms the Schiff base, which is rearranged into the stable Amadori product and further generates the stable substance by the continuous reaction of the Amadori product [36]. Separated at 15.585 min, the quasi-molecular ion peak shown in Figure 5B was obtained at m/z 365.24. The possible chemical formula was C_20_H_30_O_6_, which was speculated to be furan-containing polymers. Similarly, as shown in Figure 5C, the quasi-molecular ion peak was obtained at m/z 502.29, separated at 14.272 min. The possible chemical formula was C_30_H_40_NNaO_4_, which was speculated to be pyrrole and pyran-containing polymers. It is difficult to determine the fine structure of the Maillard reaction product, but its unit structure could be characterized to some extent. After the thermal degradation of melanoid prepared by the glucose-glycine/glutamic acid reaction or extracted from bread, heterocyclic molecules such as furan, pyrrole, pyrazine, and pyridine were generated. Melanoid could be formed by the small subclasses through cross-linking and with active amino acids as side chains. The subclasses may have furfuryl pyrrolidone, pyrrolidene-furanone, furfuryl pyrrolidone, and other unit structures [40]. Some studies also proved that chromophore of protein-sourced melanosomes usually contained pyrrole, imidazole, and nitrogen-containing derivatives, among which the nitrogen-containing structure could cross-link with proteins; the cross-linked substances and carbohydrates could further produce a furan structure during the heating [41].

Overall, the exploration of UHPLC-MS suggested that the brown pigment consisted of various chemical compounds containing glycosylation products, polymers composed of units of pyrrole and furan. The presence of these compounds in the brown pigment might be derived from the Maillard reaction. However, few studies on the color of Beijing roast duck have been conducted until now. Further research is needed to identify the molecular structure of brown pigment to clarify the browning mechanism.

## 4. Conclusions

In summary, this work illustrated the practical application of a W/O nanoemulsion to enhance the color tone of Beijing roast duck. The fabricated W/O nanoemulsion, with smaller droplet size and desirable stability, could be evenly distributed on the duck skin, thus improving the degree of the Maillard reaction, color tone, and browning for Beijing roast duck. In addition, the W/O nanoemulsion based on GRAS materials loaded with natural active compounds shows promise for decreasing the amount of added sugar. More stable nanoemulsions with less added sugar remain to be further investigated. This study provides new ideas for increasing applications of W/O-nanoemulsion-based Maillard reaction in the roast food industry, especially for the development of reduced-sugar Beijing roast duck with uniform and desired color.

## Data Availability

Not applicable.
